# Dysregulated Urinary Arginine Metabolism in Older Adults With Amnestic Mild Cognitive Impairment

**DOI:** 10.3389/fnagi.2019.00090

**Published:** 2019-04-24

**Authors:** Yue-qi Zhang, Ya-bin Tang, Eric Dammer, Jian-ren Liu, Yu-wu Zhao, Liang Zhu, Ru-jing Ren, Hong-zhuan Chen, Gang Wang, Qi Cheng

**Affiliations:** ^1^Department of Neurology & Neuroscience Institute, Ruijin Hospital affiliated to Shanghai Jiao Tong University School of Medicine, Shanghai, China; ^2^Department of Pharmacology and Chemical Biology, Shanghai Jiao Tong University School of Medicine, Shanghai, China; ^3^Department of Biochemistry, Center for Neurodegenerative Diseases, Emory University School of Medicine, Atlanta, GA, United States; ^4^Department of Neurology, Shanghai Ninth People’s Hospital, Shanghai Jiao Tong University School of Medicine, Shanghai, China; ^5^Department of Neurology, Shanghai Sixth People’s Hospital, Shanghai Jiao Tong University School of Medicine, Shanghai, China; ^6^School of Public Health, Shanghai Jiao Tong University, Shanghai, China

**Keywords:** mild cognitive impairment, urine, arginine, biomarker, early diagnosis

## Abstract

**Background**: Urine samples, which capture an individual’s metabolic profile, are ideal for the exploration of non-invasive biomarkers to confirm the amnestic mild cognitive impairment (aMCI) status of patients vs. unimpaired ones.

**Objective:** We aimed to detect differentially metabolized amino acids, which are important objectives in metabolomics, garnering particular attention in biomedical pathogenesis from the urine of aMCI patients, which may give clinicians the possibility to intervene with early treatments that curb Alzheimer’s disease (AD).

**Methods**: The study included 208 subjects, 98 of whom were aMCI patients, and 110 who were control subjects without dementia. Urine samples were taken from each participant and supernatant was obtained for analysis. The concentrations of amino acids were measured by liquid chromatography-tandem mass spectrometry (LC-MS/MS).

**Results**: Urinary arginine levels in aMCI patients are obviously lower than in normal controls (*q* < 0.2 and *p* < 0.05). Meanwhile, aMCI patients had significant reduced urinary global arginine bioavailability ratio (GABR), and GABR in urine displayed a positive correlation with the score of CMMSE.

**Conclusion**: Urinary dysregulated arginine metabolism that may serve as a helpful clinical diagnostic biomarker for aMCI in older adults.

## Introduction

Mild cognitive impairment (MCI) represents an intermediate state of cognitive function that is between the level of cognition seen in healthy aging and the severely diminished capacity for cognition reflected in criteria for dementia, of which, Alzheimer’s disease (AD) is the most prevalent in those over the age of 65 (Petersen et al., 1999). The prevalence of MCI among older adults aged 65 and over has been estimated to range from 10% to 20% (Petersen, 2011), and an increase in incidence is inevitable as the proportion and absolute numbers of the population over 65 increase rapidly. Clinically, MCI can be classified into two subtypes: amnestic and non-amnestic (Petersen, 2004). Amnestic MCI (aMCI) is characterized by prominent forgetfulness but relative preservation of other cognitive abilities, such as executive function, and visuospatial skills. To date, several studies have shown that elders with MCI are more likely to develop AD at follow-up (Busse et al., 2006; Petersen and Negash, 2008), especially aMCI (Manly et al., 2008), compared to those who are elderly without MCI. Accordingly, aMCI is increasingly recognized to be an early stage of AD and represents a therapeutic window within which to intervene more effectively, preventing potentially irreversible further loss of cognition. Therefore, studies to identify biomarkers for AD should focus on MCI or prodromal states of AD, especially aMCI.

Although several studies have identified the predictive ability of some biomarkers in cerebral spinal fluid (CSF) to predict an individual’s likelihood of converting to AD (Shaw et al., 2007; Hampel et al., 2009; Mattsson et al., 2016), a noninvasive biomarker that can assist in diagnosis of aMCI would be more widely implementable and of great value. Unlike CSF assessments, however, measurements from urine, which capture an individual’s metabolic profile, can be collected noninvasively and are less likely to be volume-limited. Because urine is not under homeostatic regulation, containing excretion of waste from many upstream metabolic processes, it can harbor abundant signatures of metabolic dysregulation, and provide insights into system-wide changes in response to physiological challenges or disease processes (Want et al., 2010). Therefore, urine samples are ideal for exploration of non-invasive biomarkers to confirm aMCI status of patients vs. unimpaired ones.

Meanwhile, liquid chromatography-tandem mass spectrometry (LC-MS/MS)-based metabolomics approaches have been developed to provide two complementary modes of analysis: targeted and untargeted modes. Untargeted metabolomics allows a broad range of both known and unknown metabolites to be surveyed for relative levels in different samples *via* particular ion intensities, whereas targeted metabolomics identifies and quantifies a specific set of known compounds, typically *via* the use of internal standards, and usually provides higher sensitivity and better dynamic range (Fiehn, 2002; Quinones and Kaddurah-Daouk, 2009).

Measurement of amino acids is an important objective in metabolomics, garnering particular attention in biomedical pathogenesis research. This is primarily because amino acids play an essential role in various biological reaction of human body, especially in neurotransmission, receptor function and neurotoxicity (Samakashvili et al., 2011), which is involved in molecular pathogenesis and development of multiple neurodegenerative diseases. Biochemical processes accounting for neurodegeneration are unclear but are likely to include the metabolism of amino acids (Fonteh et al., 2007; Hasegawa et al., 2012; Cui et al., 2014; Kori et al., 2016). Consequently, alteration of amino acid metabolism could be a precursor if not also an indicator of neurodegeneration in AD.

Previous studies mainly focused on neurotransmitter pathways or body fluid profiles. The untargeted metabolomics study from Cui et al. (2014) found Glutamine and 5-L-glutamylglycine were two metabolites in urine samples that could clearly differentiate healthy controls and AD patients. Another study has shown that homocysteic acid levels positively correlate with MMSE score (i.e., they decrease in individuals with lower cognition) in urine from AD subjects (Hasegawa et al., 2012). However, studies focused on all essential amino acids in aMCI elders are rare. Herein, a more comprehensive and accurate study to determine altered amino acids in aMCI *via* targeted metabolomics was performed, from which we expected the discovery of biomarker(s) with predictive power that also may yield conclusions indicative of biochemical changes in the general aMCI population.

## Materials and Methods

### Subjects

A total of 208 community-dwelling elderly individuals recruited from the city of Shanghai in this study were selected as a discovery set, with 98 aMCI patients and 110 healthy volunteers of comparable age and sex included as controls. A second independent set of 26 aMCI and 26 controls for validation of metabolic findings were collected from the memory clinic of hospital. All participants were aged at least 60 years. No concomitant medication intended to influence urine amino acids levels was permitted. The study was approved by the Research Ethics Committee, RuiJin Hospital affiliated to Shanghai Jiao Tong University School of Medicine, China. Written informed consent was obtained from each participant. All human research was performed in accordance with Chinese code of ethical conduct. It took 1 year to collect all samples.

### Assessment of Cognitive Impairment

The aMCI patients were diagnosed following Peterson clinical criteria originally proposed by Petersen et al. (1999). All the subjects had complaints of memory loss either self-reported or reported by their family members, but no impairment of daily activities and no dementia were reported. Longitudinal neuropsychological tests of memory showed objective impairment (≥1.5 SD below the age-appropriate mean) and no significant functional decline. MMSE and clinical dementia rating (CDR) were selected to describe the cognitive status of aMCI subjects. All the tested subjects reached 0.5 in CDR score. In addition, all aMCI patients underwent imaging and laboratory tests to confirm the absence of other possible pathologies underlying the symptoms. Control subjects were evaluated by a neurologist to confirm that there was no history of dementia or other neurologic diseases. Cognitive performance was assessed according to the Chinese version of the Mini-Mental State Examination (CMMSE). The CMMSE was translated from the modified version of the Mini-Mental State Examination (MMSE) with some items rephrased using culturally appropriate wording based on sociocultural differences of the Chinese population (Zhang et al., 1990) so that the instrument was more suited to understanding by Chinese elderly persons. This study adapted the cutoff points according to the educational level of respondents, as have previous studies in China (Cui et al., 2011). In addition, renal function and serum electrolyte were determined to be normal in all participants by laboratory examination.

### Urine Collection and Pretreatment

All urine specimens for urinalysis were obtained by the morning mid-stream clean catch method, collected under aseptic conditions. Urine was collected in sterile containers from all participants, stored in an ice bag temporarily, and then taken back to the lab for pretreatment. The individuals did not eat or drink anything in the morning before urine specimens collection. All samples were centrifuged at 17,949× *g* at 4°C for 10 min. The supernatant was frozen at −80°C until LC-MS/MS analysis.

### Measurement of Amino Acids by LC-MS/MS

#### Chemicals

All AA standards, HPLC-MS grade formic acid, and ammonium formate were purchased from Sigma Aldrich (St. Louis, MO, USA). Methanol, isopropanol, acetonitrile and water for HPLC were of LC-MS grade and obtained from Merck KGaA (Darmstadt, Germany).

#### Amino Acid Extraction

Phenylalanine-d5 was selected as the internal standard (IS) for its corresponding amino acid. All IS were dissolved in acetonitrile to yield a final concentration of 100 ng/mL. One-hundred microliters of urine was then mixed with 200 μL IS solution, and vortexed for 1 min. Then, fifty microliters of this mixture was added into 950 μL water and further vortexed for 1 min. Following centrifugation at 13,000× *g* for 5 min at 4°C, the supernatant was transferred to an autosampler vial for LC-MS/MS analysis.

#### Liquid Chromatography and Mass Spectrometry

An HPLC-quadrupole-time of flight hybrid mass system, which consisted of a LC-20AD UFLC system (Shimadzu, Kyoto, Japan) and an API4000 Qtrap triple quadrupole time of flight mass spectrometer (SCIEX, Framingham, MA, USA), was used for quantitative amino acid analysis. Chromatographic separation was performed on a ZORBAX RRHD (Rapid Resolution High Definition) Eclipse XDB 80 Å C18, 2.1*50 mm, 1.8 μm, 1,200 bar UPLC column (Agilent, Santa Clara, CA, USA) and subjected to gradient elution. The mobile phase consisted of solvent A (acetonitrile) and solvent B (water with 0.3% formic acid). The column oven was maintained at 40°C. The following gradient elution was used at a flow rate of 200 μL/min (Panel A) and 350 μL/min (Panel B): 0–1 min: 60% B → 60% B; 1–4 min: 60% B → 40% B; 4–5 min: 40% B → 40% B; 5–5.01 min: 40% B → 60% B, and 5.01–6 min: 60% B. The eluents were monitored in positive electrospray ionization (ESI) mode. The ionization source parameters were as follows: turbo heater temperature 600°C, nebulizer gas (GAS1) 60 psi, turbo heater gas (GAS2) 60 psi, curtain gas 35 psi, ion-spray voltage 5.5 kV, and collision gas for collision-induced dissociation (CID) 10 psi. The collision energy (CE) and declustering potential (DP) were 20 eV and 80 V, respectively (Table 1). The LOD (limit of detection) was defined as the lowest detectable concentration with a signal-to-noise (S/N) ratio of 3 for the LOD and 10 for the LOQ (limit of quantification). For Panel A (leucine, alanine, valine, isoleucine and glutamic acid), the seven calibrator concentrations 1, 5, 10, 50, 100, 500, and 1,000 ng mL^−1^ were evaluated, whereas the concentrations 10, 50, 100, 500, 1,000, 5,000, and 10,000 ng mL^−1^ were studied with respect to Panel B (cysteine, arginine, proline, methionine, phenylalanine, tryptophan, serine, threonine, tyrosine, glycine, glutamine, asparagine, aspartic acid, lysine, and histidine). Quality-control (QC) samples were prepared in a similar way but independently from the calibrators. Three levels of concentration were set. The analytes of Panel A included 2.5 ng mL^−1^ for low-QC samples, 40 ng mL^−1^ for medium-QC samples, and 800 ng mL^−1^ for high-QC samples. For Panel B, the QC sample concentrations were 25 ng mL^−1^, 400 ng mL^−1^, and 8,000 ng mL^−1^ at low, medium, and high levels, respectively. The QC samples were stored at −70°C. The method was validated with respect to linearity, accuracy, precision with QC samples. The LOD, LOQ, retention time and correlation coefficient (*R*^2^) of each analyte are listed in Table 1 and Supplementary Figure S1.

**Table 1:** Multiple reaction monitoring parameters for amino acid targeted metabolomics.

Analytes	Precursor ion (m/z)	Product ion (m/z)	Collision energy	LOD (ng/mL)	LOQ (ng/mL)	Retention Time (min)	*R*^2^
Cysteine	122.2	76.1	20	10	20	2.94	0.9951
Arginine	175.2	70.1	28	50	100	4.86	0.9856
Leucine	132.1	86.1	15	50	100	2.38	0.9996
Proline	116.1	70.1	20	10	20	2.25	0.9974
Alanine	90.1	44.1	15	50	100	1.71	0.9902
Valine	118.1	72.1	13	50	100	1.81	0.9998
Methionine	150.1	104.1	15	10	20	2.01	0.9976
Phenylalanine	166.2	120.1	19	10	20	1.30	0.9978
Tryptophan	205.2	146.1	25	50	100	1.74	0.9982
Isoleucine	132.1	86.1	15	50	100	2.11	0.9996
Serine	106.1	60.1	18	50	100	3.61	0.9906
Threonine	120.1	74.1	18	50	100	3.26	0.9984
Tyrosine	182.5	136.1	19	50	100	2.64	0.9976
Glycine	76.1	30.1	16	200	500	3.51	0.9826
Glutamine	147.1	84.1	24	200	500	3.52	0.9824
Asparagine	133.1	74.1	22	200	500	3.60	0.9837
Aspartic acid	134.1	74.1	22	50	100	3.52	0.9971
Glutamic acid	148.1	84.1	21	50	100	1.72	0.9964
Lysine	147.2	84.1	24	50	100	4.92	0.9948
Histidine	156.2	110.1	20	200	500	4.76	0.9833
Phenylalanine-d_5_	171.2	125.1	19			1.31^b^/2.36^a^	

*Note: ^a^ and ^b^refer to the retention time of phenylalanine-d_5_ in Panel A and Panel B analysis, respectively*.

#### Data Processing and Statistical Analysis

Data were reported as mean ± standard error of the mean (SEM). Analyst software (Version 1.5.2, AB SCIEX, Framingham, MA, USA), calculated the corresponding concentrations of amino acids relative to known amounts of IS. All statistical analyses were performed using software (SPSS Statistics for Windows; SPSS, Chicago, IL, USA). Group characteristics were compared by using *χ*^2^ (for categorical variables) and independent-sample two-sided *t*-tests (for continuous variables). A significant difference was inferred when a *P*-value was less than 0.05. We adopted FDR to account for multiple testing, considering metabolites with FDR < 0.2 and *p* < 0.05 as statistically significant in this study (Lan et al., 2011). Metabolic enrichment analysis was evaluated based on the Metaboanalyst platform^1^. The Enrichment Analysis module helps researchers identify the most relevant pathways involved in the study. We uploaded discriminatory compounds, and the built-in Homo sapiens (human) pathway library for pathway analysis was employed. Pathway enrichment analysis results were then presented graphically as well as in a detailed table.

## Results

### Demographic and Clinical Data of Community Cohort Groups

Table 2 displays the demographic and clinical data of the community cohort, with 98 aMCI and 110 control subjects. The two groups did not differ significantly from each other in age and gender. Compared with normal controls, patients of aMCI had poor cognitive performance as measured by CMMSE (*p* < 0.001).

**Table 2:** Demographic and clinical data of community cohort groups.

Subjects	aMCI	Control	*P*-value
Number of participants	98	110	
Age (mean years ± SD)	75.85 ± 6.21	76.74 ± 6.60	0.365
Male/female (n/n)	25/73	29/81	0.889
CMMSE (mean score ± SD)	20.17 ± 5.50	25.64 ± 2.73	<0.001

### Metabolomic Assessment for Urinary Amino Acids in a Community Cohort

We identified how well 20 naturally occurring amino acids distinguished between aMCI patients and normal control subjects in our community cohort (Table 3). Two amino acids detected in aMCI patients, arginine and cysteine, were significantly different from normal controls (*q* < 0.2 and *p* < 0.05), whereas other amino acid levels did not significantly differ between patients with aMCI and normal controls. The significance of difference for arginine between the two groups, with lower urinary arginine levels in aMCI patients compared to normal controls, suggests that altered arginine metabolism occurs in aMCI patients. Meanwhile, the aMCI group also demonstrated an increased concentration of cysteine, with a mean value of 230.43 ng/ml compared with 177.42 ng/ml in controls, suggesting that altered cysteine metabolism occurs in aMCI patients. Further, we examined two arginine-related metabolites, citrulline and ornithine, and calculated the urine global arginine bioavailability ratio (GABR), the ratio of arginine to its metabolites ornithine and citrulline [Arginine/(Ornithine + Citrulline)], which is a better indicator of dysregulated arginine metabolism than concentration alone. Results demonstrate that compared with normal control, aMCI patients have significantly reduced GABR (Table 3).

**Table 3:** Assessment of urine amino acids in discovery set (*n* = 208).

Amino acid	aMCI (ng/ml)	Control (ng/ml)	*q*-value	*p*-value
Cysteine	230 ± 186	177 ± 13	0.097*	0.009^#^
Arginine	481 ± 23	598 ± 36	0.097*	0.019^#^
Leucine	202 ± 10	204 ± 11	>0.999	0.666
Proline	67 ± 5	65 ± 4	>0.999	0.646
Alanine	2,155 ± 145	2,133 ± 142	>0.999	0.781
Valine	247 ± 13	247 ± 13	>0.999	0.767
Methionine	77 ± 4	72 ± 4	>0.999	0.546
Phenylalanine	430 ± 23	439 ± 24	>0.999	0.892
Tryptophan	761 ± 61	767 ± 49	>0.999	0.607
Isoleucine	132 ± 7	131 ± 7	>0.999	0.920
Serine	4,098 ± 227	3,942 ± 207	>0.999	0.525
Threonine	1,477 ± 110	1,434 ± 103	>0.999	0.577
Tyrosine	1,386 ± 102	1,418 ± 94	>0.999	0.765
Glycine	6,388 ± 480	7,627 ± 610	0.795	0.183
Glutamine	15,147 ± 889	13,685 ± 828	>0.999	0.186
Asparagine	2,224 ± 127	2,046 ± 129	>0.999	0.140
Aspartic acid	159 ± 6	176 ± 16	>0.999	0.577
Glutamic acid	297 ± 21	303 ± 24	>0.999	0.754
Lysine	2,719 ± 242	2,925 ± 393	>0.999	0.692
Histidine	27,463 ± 1,784	26,426 ± 1734	>0.999	0.619
Citrulline	150 ± 69	139 ± 88	0.305	0.088
Ornithine	395 ± 223	426 ± 291	0.208	0.046^#^
GABR	0.59 ± 0.35	0.88 ± 0.31	0.0021*	0.0005^#^

*q < 0.20, ^#^p < 0.05.

### Validation of Two Differentiable Amino Acids in aMCI Patients From the Memory Clinic

To confirm the metabolic changes of arginine and cysteine in the urine of aMCI individuals, it was tested whether the arginine and cysteine concentration of aMCI patients from a hospital memory clinic were different from those of control subjects. In this independent set, no difference was found between patients and control groups in gender and age (Table 4). Measurement was performed by LC–MS/MS according to the same method. As shown in Table 5, only the level of arginine rather than the level of cysteine in the urine of aMCI subjects was significantly lower than that of control subjects. Further, we examined two arginine-related metabolites, citrulline and ornithine, as absolute levels in memory clinic patients at the same time, and calculated the GABR, a better indicator of dysregulated arginine metabolism than concentration alone. Results demonstrate that compared with normal control, aMCI patients have significantly reduced GABR (Table 5 and Supplementary Figure S2). The results showed that aMCI patients had a significant reduced ratio, suggesting that the arginine catabolism of aMCI patients is increased, consistent with previous results from a community cohort.

**Table 4:** Demographic and clinical data of memory clinic cohort groups.

Subjects	aMCI	Control	*P*-value
Numbers of participants	26	26	
Age (mean years ± SD)	71.18 ± 10.20	77.00 ± 6.01	0.725
Male/Female (n/n)	8/18	8/18	1
CMMSE (mean score ± SD)	26.46 ± 0.27	28.31 ± 0.22	<0.001

**Table 5:** Assessment of urine amino acids in validation set (*n* = 52).

Amino acid	aMCI (ng/ml)	Control (ng/ml)	*q*-value	*p*-value
Cysteine	54 ± 52	75 ± 48	0.142*	0.141
Arginine	223 ± 134	338 ± 175	0.027*	0.011^#^
Citrulline	93 ± 68	121 ± 57	0.142*	0.125
Ornithine	252 ± 166	436 ± 326	0.027*	0.013^#^
GABR	0.48 ± 0.29	0.69 ± 0.29	0.0007*	0.0001^#^

*q < 0.20, ^#^p < 0.05.

### Levels of Arginine and GABR in Urine as a Potential Biomarker of aMCI

**Figure 1:**
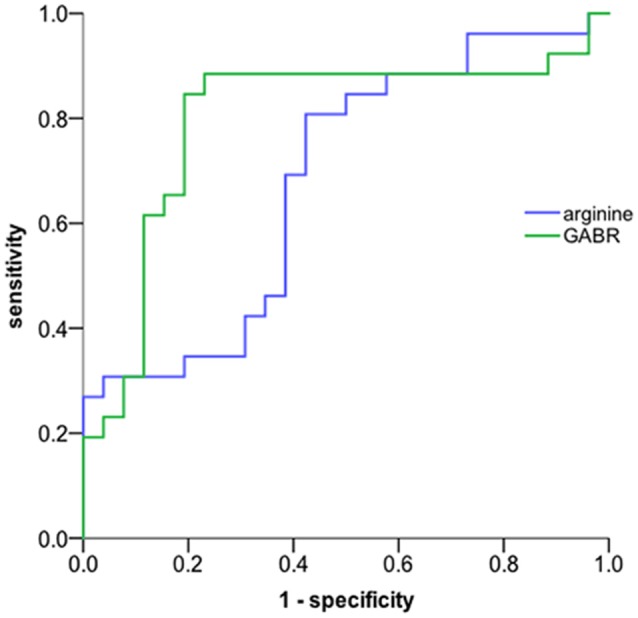
ROC curve. The global arginine bioavailability ratio (GABR) is a more sensitive diagnostic measurement compared to single concentration. The AUC of urine GABR and arginine for detection was 0.797and 0.682, respectively.

ROC analysis was performed to evaluate the potential biomarker power for classifying aMCI. In our ROC analysis differentiating between aMCI and normal control (Figure 1), the area under the curve of arginine is 0.682, the best cut-off point is 288.5 ng/mL, at which the sum of sensitivity and specificity reaches the maximal value, with a sensitivity of 80.8% and specificity of 42.3% for aMCI diagnosis; the area under the curve of GABR is 0.797, the best cut-off point is 0.523, at which the sum of sensitivity and specificity reaches the maximal value, with a sensitivity of 84.6% and specificity of 80.8% for aMCI diagnosis. This result suggests that urine GABR has a relative higher value compared to single concentration in noninvasive quantitative diagnosis of aMCI.

### Metabolic Enrichment Analysis

**Figure 2:**
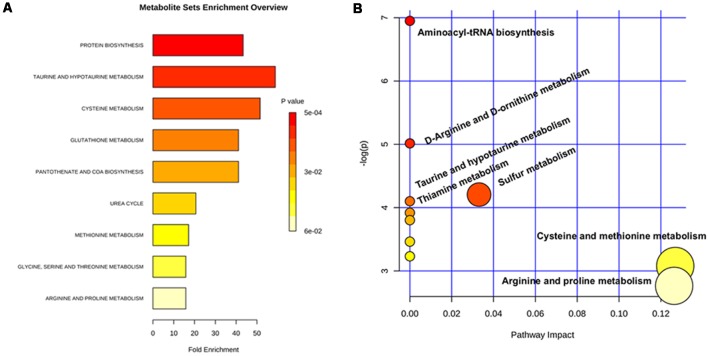
Metabolic pathway analysis based on the differentiated urine metabolites. **(A)** Overview of metabolite sets enrichment analysis based on the differentiated urine metabolites. **(B)** Urine metabolite-based metabolic pathway analysis.

A metabolic enrichment analysis demonstrated that arginine fall into pathways affected by amino acid turnover or protein biosynthesis, cysteine metabolism, urea metabolism, as well as arginine and proline metabolism (Figure 2).

### The Correlation Between GABR and Cognitive Performances

We investigated correlations between GABR and cognitive performances in 52 subjects, GABR in urine displayed a positive correlation with the score of CMMSE and suggesting that urine decreased GABR is likely related to cognitive impairment and reflecting the severity of it (Spearman correlation test, *p* = 0.031, *r* = 0.299). However, there are no correlations between arginine concentrations and CMMSE among the sample from both Community and Hospital (*p* > 0.05).

## Discussion

**Figure 3:**
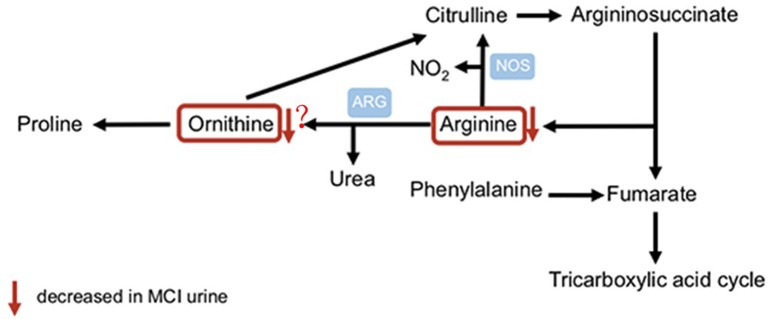
Metabolic pathways of arginine.

In the present study, we discovered and validated that arginine in urine is a potential diagnostic biomarker for aMCI. First, we identified two differentiating amino acids between aMCI patients and healthy controls from urine samples of a community cohort. Then, we validated the results from the clinic cohort and found that arginine was disturbed in aMCI patients urine. Accordingly, the present cohort plus validation study of urinary amino acids in MCI reveals evidence of dysregulated arginine metabolism that may serve as a helpful clinical diagnostic biomarker for aMCI. According to our present study and those of others (Liu et al., 2014; Wang et al., 2014), we propose that altered urinary arginine levels might reflect systematic dysregulated arginine metabolism. The changes in concentrations of arginine reflect a specific pathogenic aspect in an early phase of progression to manifest AD (Figure 3).

The role of altered arginine metabolism involved in pathology and pathophysiology of neurodegeneration has been debated: (1) with regard to the postmortem analysis of brains from AD patients, a marked increase in total arginase activity was observed compared to the control (Liu et al., 2014); (2) in plasma, there are controversies about arginine with disease progression. Several investigations showed there is a significant increase in arginine with cognitive decline (Ravaglia et al., 2004; González-Domínguez et al., 2014; Graham et al., 2015). Another recent metabolomic analysis of successful cognitive aging found lower levels of serum arginine in participants with superior memory (Mapstone et al., 2017); (3) in CSF, there are also controversial findings. In one study by Ravaglia et al. (2004), there was found a significant elevation in arginine concertation of aMCI and AD patient CSF. However, in other cohort studies, a slight decrease in arginine was detected in CSF of AD patients (Fonteh et al., 2007; Ibáñez et al., 2012); and (4) with regard to urine, only one study revealed that urine arginine concentration was higher in probable AD than control participants but did not find a statistical difference (Fonteh et al., 2007), and to our knowledge, there are no data about urinary arginine of MCI individuals published to date.

In the present study, we found significantly decreased urinary arginine levels for aMCI individuals. The ratio of arginine to ornithine and citrulline is considered to be a better indicator of dysregulated arginine metabolism than concentration alone, the reduced ratio suggests elevated consumption of arginine in individuals with aMCI. There is much evidence suggesting that arginine may play a prominent role in the pathogenesis of diverse age-related diseases, and could be required for memory consolidation (Rayatnia et al., 2011). Sustained arginine deprivation may lead to cell death *in vitro* (Kuma and Mizushima, 2010). Additionally, it is notable that an increase in bioavailable arginine negatively associates with water-maze performance as rats become aged (Cassel et al., 2005). Moreover, some previous studies of AD mouse models and patients also support that the supplementation of arginine could improve memory consolidation and learning (Calabro et al., 2013; Wei et al., 2013). In sum, these findings suggest that there is a likely relationship between dysregulated arginine metabolism and impairment in the maintenance of cognition.

The discriminatory metabolites that were elucidated in this study, namely decreased urinary arginine, are also involved in related biochemical pathways inside the body, especially taurine and hypotaurine metabolism, which plays a neuroprotective role and may be protective against excitotoxicity (Wu et al., 2008). Moreover, glutathione (GSH), the most abundant endogenous antioxidant in the brain, is decreased in AD and aging. A decrease of antioxidants, particularly reduced GSH, may be a major contributor to the progression of MCI to AD (Baldeiras et al., 2010). This in combination with our findings suggests that altered arginine level may follow from altered protein homeostasis, taurine metabolism, glutathione metabolism, or many other metabolic pathways that closely relate to the AD pathological process.

We also found that GABR may be a more sensitive diagnostic measurement compared to a single concentration of arginine. In our ROC analysis, the AUC of GABR is 0.797, the sum of sensitivity and specificity reaches the maximal value when the sensitivity of 0.846 and specificity of 0.808 for aMCI diagnosis. Recently, GABR has been proposed as a marker of nitric oxide synthetic capacity (Tang et al., 2009), which is decreased in posttraumatic stress disorder (PTSD) and correlated with symptom severity and markers of inflammation (Bersani et al., 2016). In addition, those with type 2 diabetes had a significantly lower GABR than individuals without diabetes (Sourij et al., 2011). Our present studies revealed a positive correlation between the value of GABR and the score of CMMSE, and the AUC of GABR is fairly noticeable. Although, more evidence is required to better demonstrate the relationship between GABR and cognitive decline in aMCI patients. However, it seems that GABR is expected to be a subtle marker to identify patients with suspected cognitive impairment.

Limitations of the present study included the following: first, we only investigated the cross-sectional status of patients without longitudinal follow-up. Meanwhile, this study only focused on aMCI samples, not including AD patient urine samples. Second, the number of samples in the validation set is less than that in the training set. Therefore, the targeted amino acids concentrations in the validation set could be different from that of the training set. Third, the urinary composition may be more apt to affected by the source of diets. In general, our present study is an important pilot study. In the future, a more powered, longer-running clinical study is required to confirm the findings presented here that urine arginine and GABR can serve as biomarkers for AD-related cognitive decline.

## Ethics Statement

The study was approved by the Research Ethics Committee, RuiJin Hospital affiliated to Shanghai Jiao Tong University School of Medicine, China. Written informed consent was obtained from each participant. All human research was performed in accordance with Chinese code of ethical conduct. It took 1 year to collect all samples.

## Author Contributions

GW, QC and HC conceived, designed and oversighted the community cohort experiments, and revised the manuscript. YZhan and YT carried out experiments, performed statistical analyses, and wrote the manuscript. GW, JL and YZhao conceived, designed and oversighted the clinic cohort experiments. RR, ED and LZ conceived, designed and oversighted community cohort experiments, performed statistical analyses, and helped draft the manuscript. All authors read and approved the final manuscript.

## Conflict of Interest Statement

The authors declare that the research was conducted in the absence of any commercial or financial relationships that could be construed as a potential conflict of interest.

## Footnotes

^1^http://www.metaboanalyst.ca/

## Supplementary Material

FIGURE S1 Representative MRM chromatograms of spiked sample. The upper refers to Panel A (leucine, alanine, valine, isoleucine, glutamic acid and phenylalanine-d5), the lower refers to Panel B (cysteine, arginine, proline, methionine, phenylalanine, tryptophan, serine, threonine, tyrosine, glycine, glutamine, asparagine, aspartic acid, lysine, histidine, and phenylalanine-d_5_).

FIGURE S2 Box plots of the distribution of arginine, cysteine, citrulline and ornithine. **p* < 0.05.
