# Supplementary figures and images for: Dysregulated Urinary Arginine Metabolism in Older Adults With Amnestic Mild Cognitive Impairment
Source: Front Aging Neurosci. 2019 Apr 24;11:90. doi: 10.3389/fnagi.2019.00090 (PMC6492563; doi:10.3389/fnagi.2019.00090)

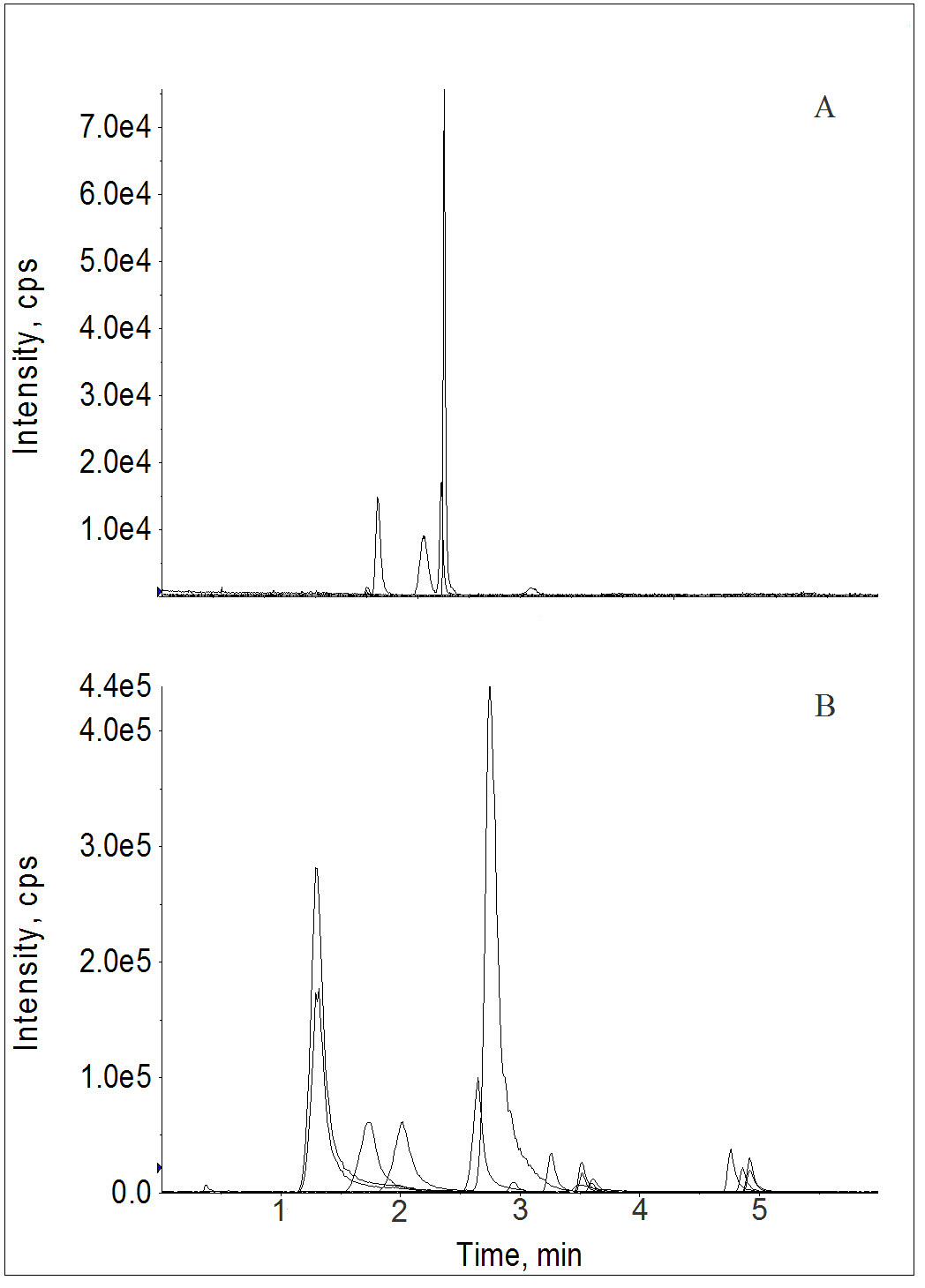

Supplement: FIGURE S1 — Representative MRM chromatograms of spiked sample. The upper refers to Panel A (leucine, alanine, valine, isoleucine, glutamic acid and phenylalanine-d5), the lower refers to Panel B (cysteine, arginine, proline, methionine, phenylalanine, tryptophan, serine, threonine, tyrosine, glycine, glutamine, asparagine, aspartic acid, lysine, histidine, and phenylalanine-d5). [file Image_1.JPEG]

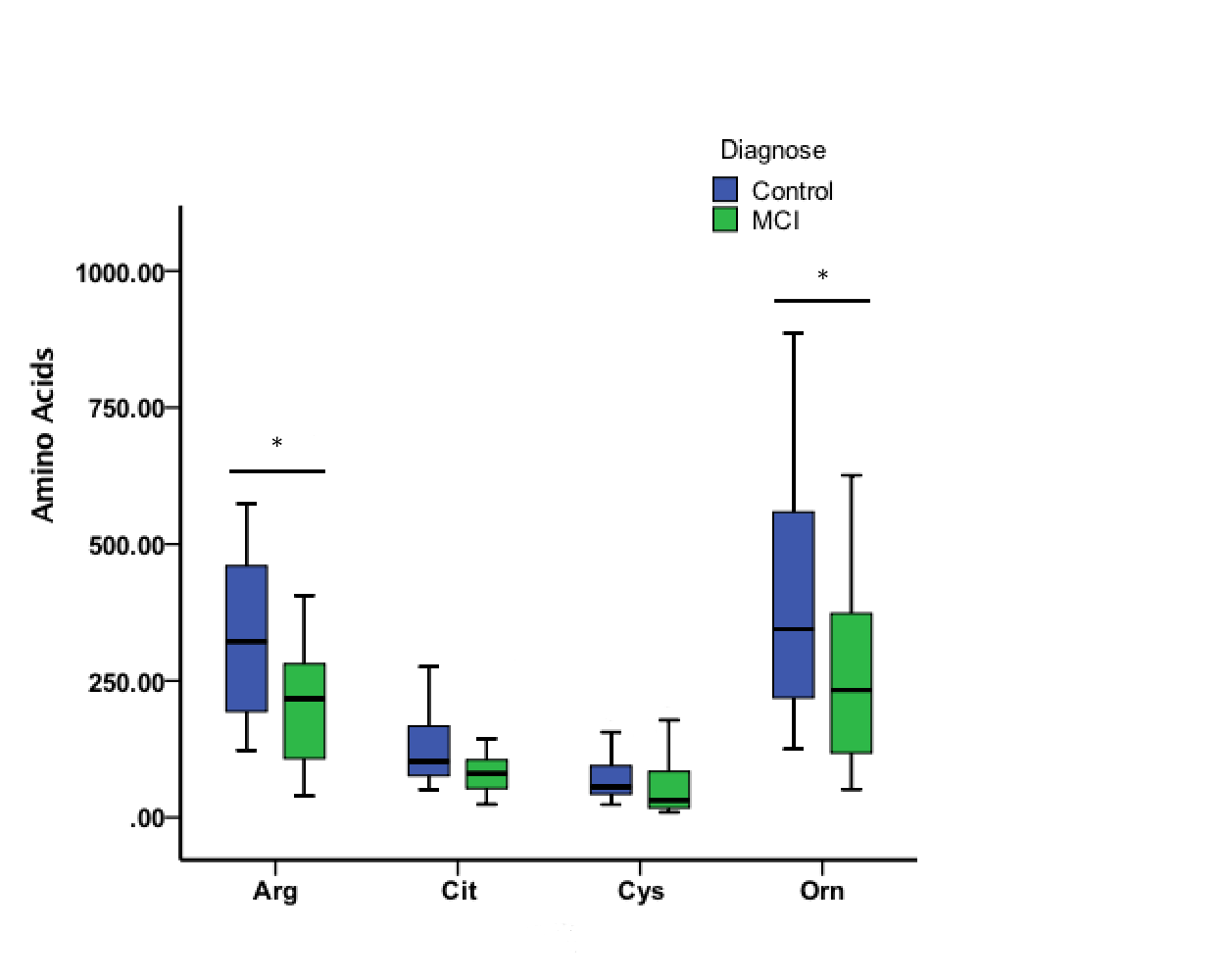

Supplement: FIGURE S2 — Box plots of the distribution of arginine, cysteine, citrulline and ornithine. *p < 0.05. [file Image_2.TIF]
